# Light-Induced Halide Segregation in 2D and Quasi-2D Mixed-Halide Perovskites

**DOI:** 10.1021/acsenergylett.3c00160

**Published:** 2023-03-03

**Authors:** Kunal Datta, Alessandro Caiazzo, Michael A. Hope, Junyu Li, Aditya Mishra, Manuel Cordova, Zehua Chen, Lyndon Emsley, Martijn M. Wienk, René A. J. Janssen

**Affiliations:** † Molecular Materials and Nanosystems, Institute of Complex Molecular Systems, Eindhoven University of Technology, P.O. Box 513, 5600 MB Eindhoven, The Netherlands; ‡ Institut des Sciences et Ingénierie Chimiques, Ecole Polytechnique Fédérale de Lausanne, Lausanne 1015, Switzerland; § Materials Simulation and Modelling and Center for Computational Energy Research, Department of Applied Physics, Eindhoven University of Technology, P.O. Box 513, 5600 MB Eindhoven, The Netherlands; ∥ Dutch Institute for Fundamental Energy Research, 5612 AJ Eindhoven, The Netherlands

## Abstract

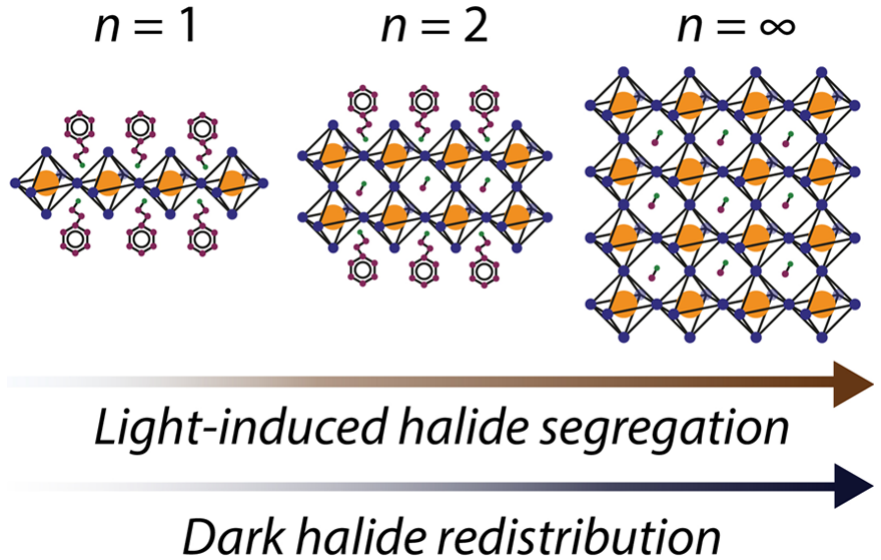

Photoinduced halide segregation hinders widespread application of three-dimensional (3D) mixed-halide perovskites. Much less is known about this phenomenon in lower-dimensional systems. Here, we study photoinduced halide segregation in lower-dimensional mixed iodide-bromide perovskites (PEA_2_MA_*n*–1_Pb_*n*_(Br_*x*_I_1–*x*_)_3*n*+1_, with PEA^+^: phenethylammonium and MA^+^: methylammonium) through time-dependent photoluminescence (PL) spectroscopy. We show that layered two-dimensional (2D) structures render additional stability against the demixing of halide phases under illumination. We ascribe this behavior to reduced halide mobility due to the intrinsic heterogeneity of 2D mixed-halide perovskites, which we demonstrate via ^207^Pb solid-state NMR. However, the dimensionality of the 2D phase is critical in regulating photostability. By tracking the PL of multidimensional perovskite films under illumination, we find that while halide segregation is largely inhibited in 2D perovskites (*n* = 1), it is not suppressed in quasi-2D phases (*n* = 2), which display a behavior intermediate between 2D and 3D and a peculiar absence of halide redistribution in the dark that is only induced at higher temperature for the quasi-2D phase.

Wide-bandgap mixed-halide perovskite absorbers find application in efficient multijunction solar cells where their tunable bandgap allows them to be combined with a variety of narrow-bandgap semiconductors (c-Si, CIGS, perovskite, organic) to increase the conversion efficiency.^1−4^ However, conventional three-dimensional (3D) perovskite systems are vulnerable to environmental factors, such as oxygen and moisture, which can compromise long-term stability.^5,6^ In addition, photoinduced halide segregation occurs in mixed-halide compositions due to the thermodynamically favorable formation of low-energy iodide-rich regions under illumination, aided by defect migration processes that can further exacerbate device instability.^7−12^

In recent years, layered Ruddlesden–Popper (RP) perovskites have shown promise as a potentially stable alternative to conventional 3D perovskite-based solar cells, light emitting diodes, and photodetectors.^13−15^ Such lower-dimensional RP systems can be visualized as a 3D perovskite sliced along its (100)-oriented crystallographic planes to form structures where a large organic spacer molecule (for example, BA^+^, butylammonium, or PEA^+^, phenethylammonium) separates slabs of conjoined lead halide octahedral sheets, intercalated with smaller monovalent organic cations (for example MA^+^, methylammonium, FA^+^, formamidinium, or Cs^+^, cesium).^16^ These lower-dimensional perovskite phases are labeled by an *n*-value, defined by the number of conjoined lead halide octahedral sheets that form each slab; *n* = 1 refers to a two-dimensional (2D) phase with independent sheets of lead halide octahedra separated by the organic spacer, *n* = 2 refers to a quasi-2D phase consisting of two conjoined octahedral sheets in each slab, and so on. By engineering the crystallization of different structural phases, the physical properties of perovskite thin films, such as the absorption spectrum, phase purity, or defect concentration, can be tuned and therefore used to modulate optoelectronic properties and material stability.^17^

In previous studies, pure-iodide or pure-bromide lower-dimensional perovskites have been successfully used to limit charge-carrier recombination at interfaces of the 3D perovskite with charge-transport layers.^18−22^ In contrast, the use of lower-dimensional mixed-halide perovskites remains a largely unexplored area.^23^ Among the studies published on such materials, it has been shown that the spacer cation may determine ion migration characteristics in mixed-halide compositions, with BA-based perovskites being more prone to halide demixing than PEA-based analogues.^24^ Furthermore, a reduced perovskite dimensionality has been correlated to suppressed ion migration, indicating the potential of 2D perovskites to prevent photoinduced instability.^25−28^ Nevertheless, the direct effect of perovskite dimensionality on instabilities inherent to mixed-halide 3D perovskite compositions is not yet firmly established.

Herein, the photostability of mixed-halide 2D perovskite (*n* = 1) systems is studied using time-dependent photoluminescence (PL) spectroscopy to identify their susceptibility to light-induced halide segregation. The photostability is then correlated with the nonbinomial halide distribution determined by solid-state ^207^Pb nuclear magnetic resonance (ss NMR). Thereafter, this behavior is studied in mixed-halide, quasi-2D perovskite thin films where the distribution between different structural phases (*n* = 1, 2, etc.) is controlled by a solvent engineering approach. This allows to correlate the tendency to undergo light-induced halide segregation with the dimensionality of the perovskite phase. The same structural properties are also found to influence halide redistribution in the dark, thereby modulating the reversibility of light-induced segregation.

2D (*n* = 1) PEA lead halide perovskites were prepared on glass substrates via one-step room-temperature spin-coating from a *N*,*N*-dimethylformamide (DMF)-based precursor solution, leading to a film with a nominal composition PEA_2_Pb(Br_*x*_I_1–*x*_)_4_. Details regarding the layer deposition can be found in the Supporting Information. Partial substitution of iodide with bromide yields a blue-shift in the absorption onset and in the PL spectrum (Figure 1a). X-ray diffraction (XRD) confirms the structural change, as the diffraction peak at 5.4°, corresponding to the (002) plane of PEA_2_PbI_4_, shifts to lower angles upon addition of Br (Figure S1).^29^

**Figure 1:**
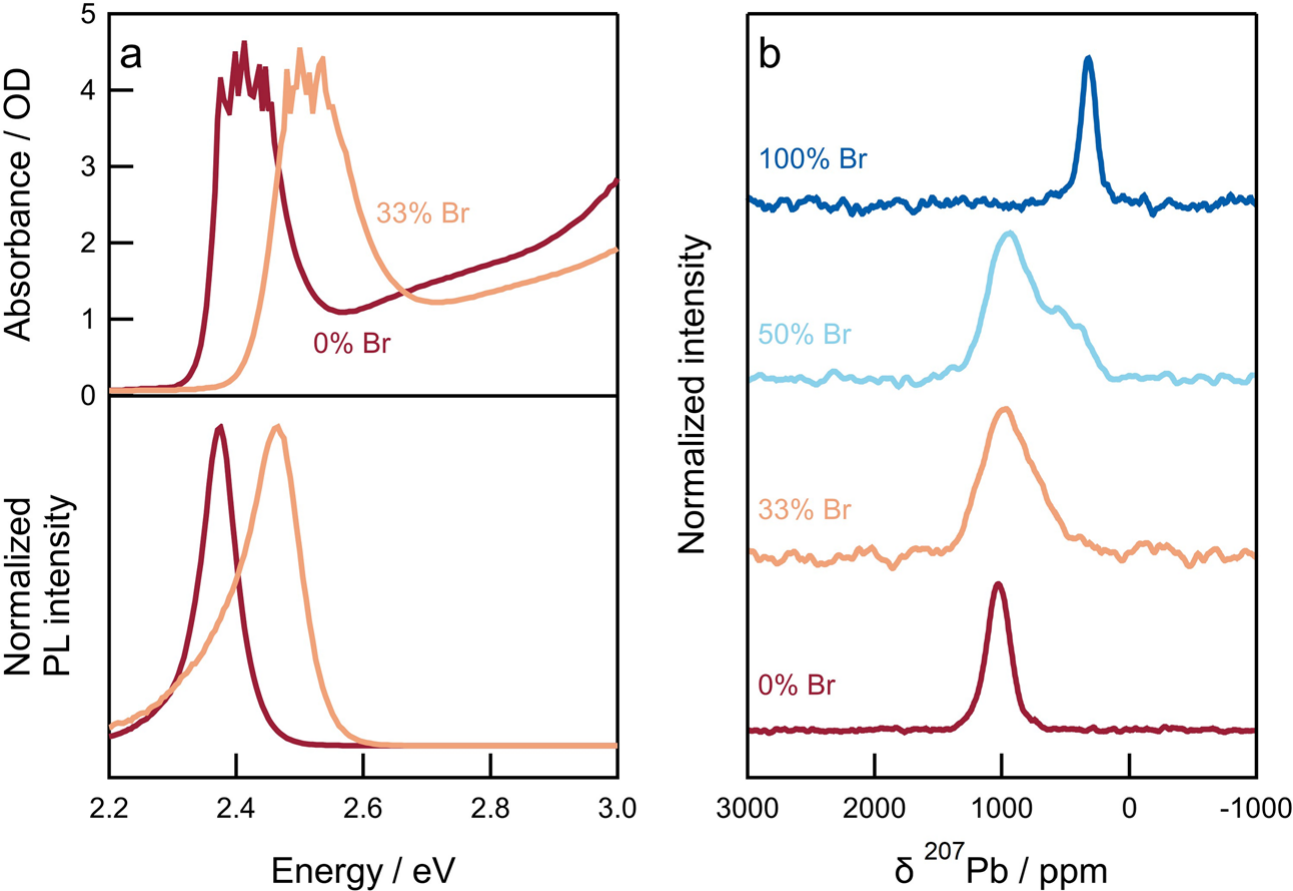
Mixed-halide two-dimensional perovskites. (a) UV–vis–NIR absorption (top panel) and steady-state photoluminescence (bottom panel) spectra of PEA_2_Pb(Br_*x*_I_1–*x*_)_4_ with *x* = 0 and *x* = 0.33. The noisy features at the top of the excitonic absorption band are an artifact and caused by the high (>4) optical density in that spectral region. (b) Isotropic ^207^Pb NMR spectra of PEA_2_Pb(Br_*x*_I_1–*x*_)_4_ for different halide compositions, obtained by summation of the corresponding phase-adjusted spinning sidebands (PASS) spectra.

^207^Pb ssNMR was performed to examine the halide distribution in PEA_2_Pb(Br_*x*_I_1–*x*_)_4_ films. Solid-state NMR has been used to study a wide variety of phenomena in halide perovskites.^30,31^ Previously, it has been shown that ^207^Pb ssNMR is sensitive to the halide ion in 3D perovskite materials and to the local configuration of the lead halide octahedra in mixed-halide perovskites.^30,32−36^^207^Pb ssNMR has also been performed for 2D and quasi-2D perovskites BA_2_MA_*n*-1_Pb_*n*_I_3*n*+1,_ showing that the ^207^Pb shift is sensitive to *n*.^37^ However, mixed-halide 2D or quasi-2D compositions have not yet been investigated.

Figure 1b shows the isotropic ^207^Pb ssNMR spectra for PEA_2_Pb(Br_*x*_I_1–*x*_)_4_ with different bromide/iodide contents (*x* = 1, 0.50, 0.33, and 0). The isotropic chemical shift for the pure-iodide PEA_2_PbI_4_ system is 1030 ppm, which is similar to the previously reported shift of BA_2_PbI_4_ (1084 ppm)^37^ but significantly lower than the shift for 3D perovskites (1265, 1445, and 1515 ppm for Cs^+^, MA^+^, and FA^+^ cations, respectively).^33,38^ Pure-bromide PEA_2_PbBr_4_ possesses a lower chemical shift of 325 ppm, consistent with the trend for 3D perovskites. Unlike the pure-iodide, the pure-bromide *n* = 1 2D perovskite is within the range of observed ^207^Pb shifts for 3D bromide perovskites (262, 365, and 515 ppm for Cs^+^, MA^+^, and FA^+^ cations, respectively).^33^

For mixed-halide 3D perovskites, different individual configurations of the lead iodide octahedra are possible, e.g., [PbI_*a*_Br_6–*a*_]^4–^ with *a* = 0–6, and the isotropic ^207^Pb chemical shift for each configuration depends approximately linearly on *a*.^32,35^ Mixed-halide 3D compositions with a 1:1 halide ratio exhibit a broad ^207^Pb resonance approximately midway between those of the corresponding pure halides, which suggests a random halide occupancy and a binomial distribution of the individual local configurations.^32,34,35,39^ Here, it can be seen that the isotropic ^207^Pb spectrum of PEA_2_PbBr_2_I_2_ (i.e., *x* = 0.50) is broader than that of the pure-iodide and pure-bromide perovskites, indicating a distribution of individual configurations rather than a single ordered arrangement. The peak in the spectrum is significantly skewed to higher chemical shift (corresponding to iodide-rich octahedra, *a* ≥ 4) but with a clear shoulder at lower chemical shift (that matches bromide-rich octahedra, *a* ≤ 2). This indicates the presence of different (not fully random) distributions of bromide and iodide across the octahedra in the sample.

The *n* = 1, *x* = 0.33 sample exhibits a similar isotropic ^207^Pb spectrum but with the center of mass shifted further to higher frequency, as expected given the higher iodide concentration. Again, the center of mass of the spectrum is at a higher frequency than what would be expected for a simple random bromide–iodide distribution over the octahedra. The observed spectra thus suggest the presence of both iodide-rich and bromide-rich octahedra in the samples. Halide heterogeneity has previously been identified in solution-processed 3D mixed-halide perovskites.^40−42^ To test this hypothesis, the isotropic chemical shielding for all 18 possible individual configurations for [PbI_*a*_Br_6–*a*_]^4–^ (with *a* = 0–6) was calculated (Table S1). The shielding again depends approximately linearly on *a* (Figure S2), as observed for the 3D perovskites. These computed shieldings were then used to simulate spectra for both random and nonrandom halide distributions (Figure S3). As expected, a random binomial halide distribution results in an approximately symmetric spectrum that does not match the experimental spectrum. In contrast, a model in which both iodide-rich and bromide-rich compositions are present affords simulated spectra that reproduce the general features of the experimental spectra. Note that the *x* = 0.5 sample is single-phase from XRD (Figure S1b), rather than segregated into iodide-rich and bromine-rich phases. Therefore, the preference for iodide-rich and bromide-rich octahedra must occur within nanoscale clusters or even at the scale of a single octahedron. Nonrandom distribution of halides at the level of a single octahedron has been demonstrated in recent studies, which have shown preferential occupation of equatorial sites for bromide ions and axial for iodides.^41,43,44^ With ssNMR, we find a nonrandom halide distribution, which may also include axial/equatorial preference within a single octahedron.

*Light-Induced Halide Segregation in Mixed-Halide 2D Perovskites*. The photostability of the mixed-halide (*x* = 0.33) film was studied by continuously illuminating the film with blue (405 nm) light (Figure S4) and tracking the evolution of the PL spectrum over time (Figure 2a,b). First, the PL spectrum is asymmetrical, which is likely due to slight compositional inhomogeneity, as observed via ssNMR. Second, the spectral centroid, during 10 000 s (approximately 3 h) of illumination at approximately 3 Sun equivalent intensity, exhibits only a marginal red-shift from 2.46 to 2.41 eV. Such a red-shift has been extensively studied in 3D perovskites and is related to the light-induced formation of iodide-rich domains which provide low-energy sites for charge-carrier recombination.^7^ However, since the PL energy never approaches that of a pure-iodide phase (2.37 eV), it can be argued that only a small proportion of low-bandgap iodide-rich phase forms in the system over time. This is in stark contrast to observations made in methylammonium-based 3D perovskites, in which a nearly pure-iodide-rich phase is formed leading to a large red-shift of the emission spectrum.^7^

**Figure 2:**
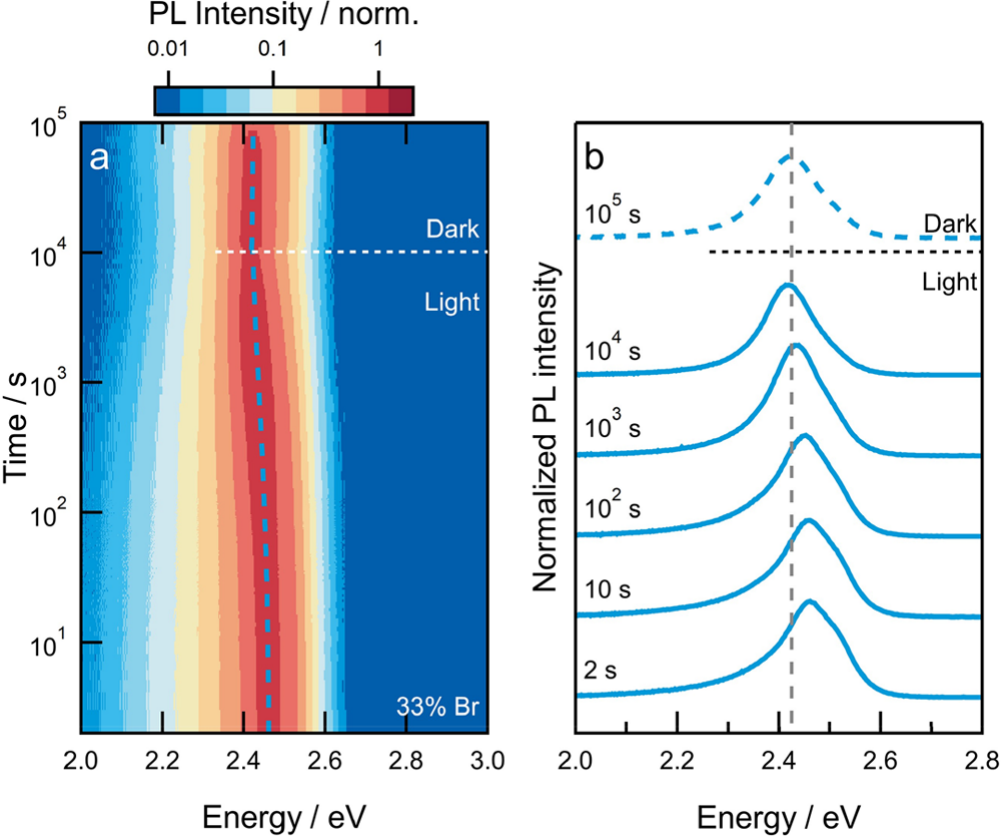
Light-induced halide segregation in mixed-halide 2D perovskite thin films. (a) Normalized PL spectra as a function of time for PEA_2_Pb(Br_0.33_I_0.67_)_4_ film. The film is illuminated by a 405 nm LED source at ∼3 Sun equivalent intensity for 10 000 s (∼3 h) followed by storage in the dark for 90 000 s (∼25 h) with spectra recorded intermittently. Note the logarithmic intensity and time axes. (b) Normalized photoluminescence spectra at selected times. The spectra are offset vertically for clarity but have the same vertical scale. The vertical dashed line in panel (b) corresponds to the emission energy at 10^5^ s (labeled Dark).

After 10 000 s of illumination, the film was stored in the dark, and, intermittently, PL spectra were recorded (light exposure for 500 ms during excitation). Under similar conditions, segregated 3D perovskites typically undergo an entropically driven redistribution of halide ions that restores the statistically mixed-halide phase leading to a blue-shift of the PL spectrum.^13^ In contrast, after storage in dark conditions for approximately 25 h, the spectrum of segregated PEA_2_Pb(Br_0.33_I_0.67_)_4_ remained steady with a maximum at 2.41 eV (Figure 2b), implying the absence of any halide redistribution.

The absence of halide segregation in the 2D perovskite can be tentatively ascribed to the nonbinomial halide distribution determined by ^207^Pb NMR. The spectra indicate a preference for iodide-rich and bromide-rich octahedra, implying an additional energy barrier for bromide ions to move into the iodide-rich octahedra and vice versa. This barrier to halide migration would suppress the segregation under light illumination as well as the recovery in the dark.

*Mixed-Halide Multidimensional Perovskites*. The very wide bandgap of 2D (*n* = 1) perovskites leads to a poor overlap of their absorption spectrum with solar irradiance and makes them less relevant for integration in solar cells. Instead, quasi-2D perovskite (2 ≤ *n* ≤ 5) systems are becoming increasingly common to balance the stability and efficiency of solar cells by combining a broad absorption profile with the stability afforded by layered structures.^9^ Mixed-halide quasi-2D (nominal *n* = 4) perovskite films were prepared using PEA as the bulky spacer and MA as the organic cationic component of the *n* > 1 phases (nominal composition PEA_2_MA_3_Pb_4_(Br_*x*_I_1–*x*_)_13_). By incorporating bromide (*x* = 0.33), the onsets of optical absorption and PL blue-shift compared to the corresponding pure-iodide (*x* = 0) quasi-2D (*n* = 4) perovskite (Figure S5), along with a shift of the Bragg peaks to larger diffraction angles, confirm the successful bromide incorporation and resulting widening of the optical bandgap.

During solution-based fabrication of quasi-2D perovskites with nominal *n* > 1, it is often the case that a distribution of structural phases with different *n-*values is obtained in the perovskite film. In most cases, quasi-2D phases with lower *n*-values crystallize at the interface with the substrate, whereas quasi-3D phases with higher *n*-values form at the interface with air. The introduction of a cosolvent (dimethyl sulfoxide, DMSO) to the DMF-based perovskite precursor solution predictably changes this structural distribution, which allows inducing stratification of the quasi-2D and quasi-3D-phases over the film thickness.^45,46^Figure 3a shows a schematic of this dimensional stratification as a consequence of cosolvent addition. For example, in films prepared from a pure DMF solvent (0% DMSO), a gradual onset of the UV–vis–NIR absorption spectrum is observed, indicating the formation of a quasi-3D perovskite phase, i.e., PEA_2_MA_*n*–1_Pb_*n*_(Br_*x*_I_1–*x*_)_3*n*+1_ with high *n-*values (Figure 3b). By increasing the DMSO fraction in the DMF precursor solution, excitonic absorption features corresponding to lower-dimensional phases appear in films at ∼2.5 eV (*n* = 1) and at ∼2.3 eV (*n* = 2). At the same time, the onset of higher-dimensional perovskite phases red-shifts with the addition of more cosolvent, leading to the formation of a 3D phase with a steep absorption onset at ∼1.78 eV when the DMSO volume concentration exceeds 10%.

**Figure 3:**
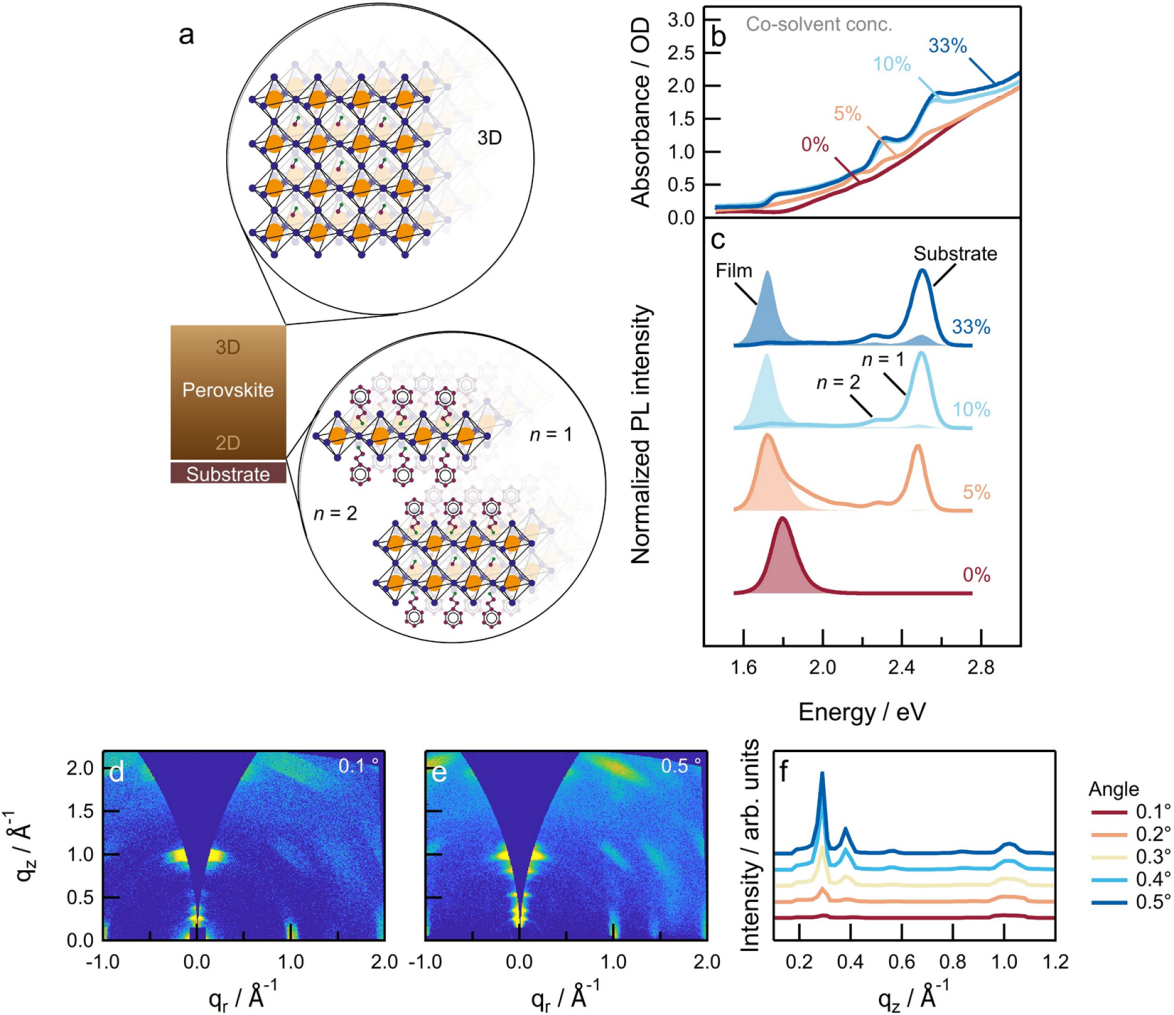
Structural stratification in mixed-halide quasi-2D PEA_2_MA_3_Pb_4_(Br_0.33_I_0.67_)_13_ perovskite. (a) Schematic of different dimensional phases (*n* = 1, *n* = 2, and 3D) formed in quasi-2D perovskite thin films by solvent engineering. (b) UV–vis–NIR absorption spectra and (c) photoluminescence spectra using excitation from the substrate side (solid lines) and film side (shaded) of perovskite films prepared using different concentrations of DMSO in the DMF precursor solutions. (d,e) Angle-resolved GIWAXS patterns of perovskite films (*n* = 4, 33% Br) prepared from a DMF precursor solution containing 20% DMSO, measured at incidence angles of (d) 0.1° and (e) 0.5°. (f) Out-of-plane (*q*_*z*_) cuts of angle-resolved GIWAXS measurements collected at incident angles of 0.1–0.5°.

PL spectra (Figure 3c) of films prepared from a DMSO-containing DMF precursor solution exhibit high-energy emissions from *n* = 1 and *n* = 2 phases when excited from the substrate side, while excitation from the film side results in a spectrum dominated by low-energy emission corresponding to an increased contribution from the 3D perovskite phase. This confirms the presence of a structural stratification, with 2D (*n* = 1) and quasi-2D (*n* = 2) phases located at the interface with the substrate and quasi-3D or 3D phases organized on top at the interface with air.^47^ Such a phase distribution is reproducible and shows a negligible sample-to-sample variability.

The stratification of 2D–3D phases is further corroborated using angle-resolved grazing-incidence wide-angle X-ray scattering (AR-GIWAXS) (Figure 3d,e). When probing PEA_2_MA_3_Pb_4_(Br_0.33_I_0.67_)_13_, raising the angle of incidence from 0.1 to 0.5° increases the penetration depth of X-rays and therefore allows characterization of the perovskite structure as a function of depth in the film. At all incident angles, the GIWAXS patterns indicate well-oriented perovskite phases with Bragg spots at *q*_*z*_ = 1 Å^–1^, corresponding to (100) planes of a quasi-3D perovskite, and at *q*_*z*_ = 0.28 and 0.38 Å^–1^, representative of (002) planes of *n* = 2 and *n* = 1 perovskites, respectively.^29,48^ At higher incident angles, the Bragg spots associated with small *n*-value perovskites increase in intensity. Analyzing the out-of-plane line cuts of the AR-GIWAXS patterns (Figure 3f) shows that these lower-dimensional phases are mostly localized at the substrate interface. In fact, the ratio (*I*_*n*__=2_/*I*_3D_) increases from 1.1 to 8.1 when going from a 0.1 to 0.5° incidence angle (Figure S6), confirming the 2D–3D stratification.

The evolution of the PL spectrum of PEA_2_MA_3_Pb_4_(I_0.67_Br_0.33_)_13_ films under continuous illumination to characterize photoinduced halide segregation in this system is shown in Figure 4. Similar to the experiment shown in Figure 1, the measurement consisted of two phases, the first phase (approximately 3 h) characterizing photoinduced PL behavior changes and the second phase (approximately 14 h) tracking the reversal of these changes in dark conditions. In order to better observe the different structural phases, films were probed from the substrate side where a higher proportion of the lower-dimensional phases is formed. To ensure that the broad distribution of different dimensional phases is excited, a combination of blue (405 nm) and green (530 nm) light was used (Figure 3a).

**Figure 4:**
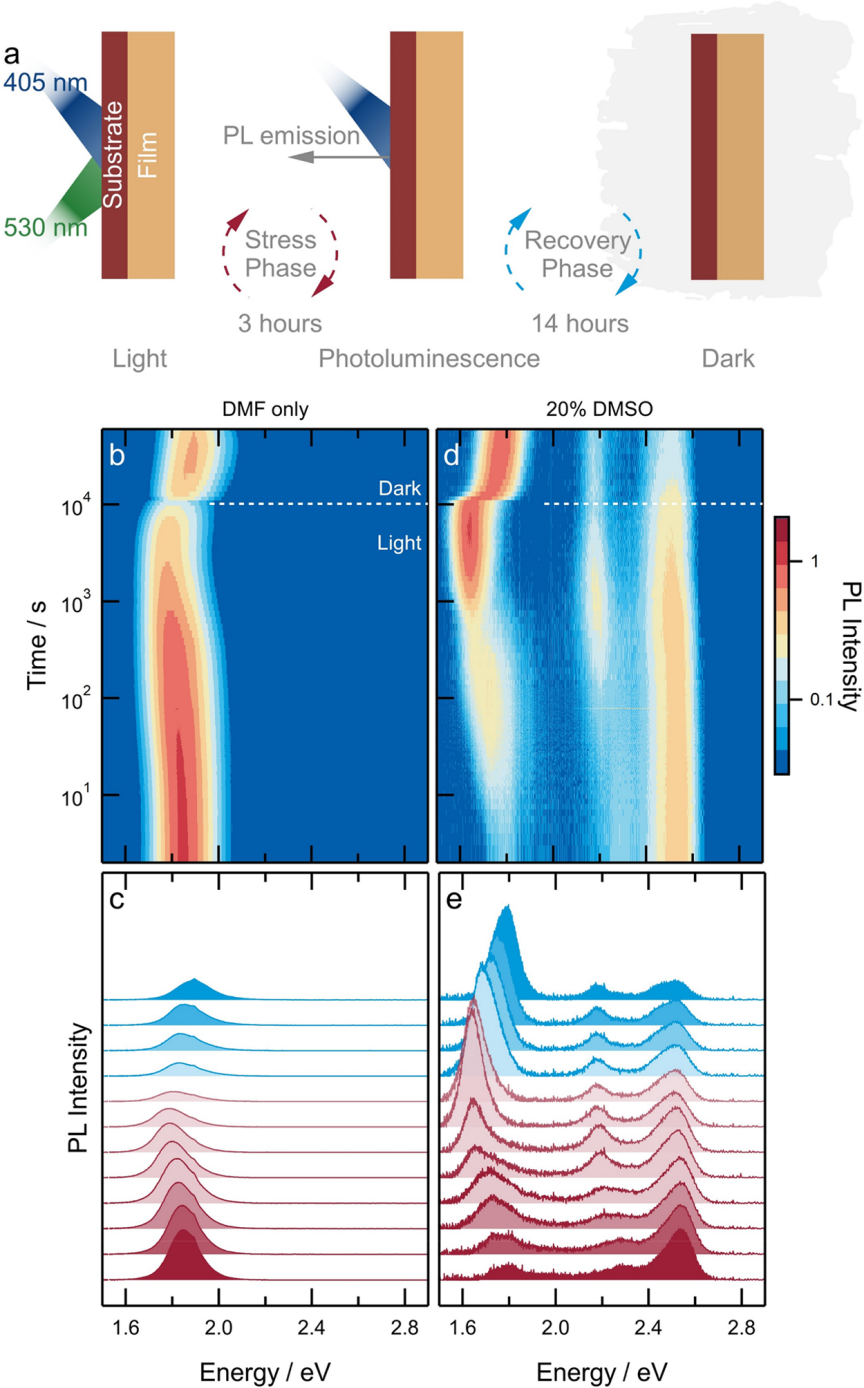
Effect of structural stratification on photoinduced halide segregation in *n* = 4 quasi-2D PEA_2_MA_3_Pb_4_(Br_0.33_I_0.67_)_13_. (a) Schematic describing the measurement protocol to characterize light-induced halide segregation and dark relaxation in mixed-halide perovskite thin films. (b–e) Normalized 2D plots of the photoluminescence intensity (b,d) and photoluminescence spectra (c,e) versus photon energy recorded over time under 10 000 s of continuous green (530 nm) and blue (405 nm) excitation, followed by 50 000 s of storage in the dark with intermittent photoluminescence measurements. Perovskite films were prepared from (b,c) DMF-only and (d,e) 20% DMSO-containing DMF precursor solutions. Note the logarithmic intensity and time axes in panels (b) and (d).

First, it was found that in an unstratified film prepared from a pure DMF solvent system, the PL spectrum corresponding to a quasi-3D perovskite appears at ∼1.84 eV and shows a mild red-shift after 1000 s of illumination. The PL intensity is also found to decrease as the film is illuminated for ∼3 h, indicating increased nonradiative recombination of photogenerated charge carriers. This behavior may result from an ion-migration-related increase in local strain, which has been shown to increase nonradiative recombination.^49^ In contrast, a film prepared with 20% DMSO in the DMF precursor solution shows PL signals corresponding to lower-dimensional phases at ∼2.5 eV (*n* = 1) and ∼2.3 eV (*n* = 2) and an emission from the 3D phase at ∼1.78 eV. Under continuous illumination, the signal corresponding to the *n* = 1 phase shows only a minor red-shift accompanied by a reduction in intensity, consistent with observations reported in Figure 1. The PL signal related to the *n* = 2 phase, which first appears as a shoulder at ∼2.3 eV, splits to form a distinct peak at ∼2.18 eV, thus indicating the formation of an iodide-rich *n* = 2 phase within ∼100 s of illumination. Similar to observations made with 3D perovskites,^8^ the red-shifted peak progressively brightens, indicating a higher radiative yield of the emission.

Lastly, the peak corresponding to a 3D phase at ∼1.78 eV also shows a red-shift and concurrent brightening within 10 s of illumination, stabilizing at ∼1.63 eV after ∼1000 s. The PL of the unstratified (nominally *n* = 4) perovskite (Figure 4b,c) is noticeably more stable with time than that of the 3D phase in a stratified film (Figure 4d,e). The influence of the cosolvent-induced structural stratification on halide segregation is also clear from other cosolvent concentrations used to prepare films. Films prepared from DMF precursor solutions with 5–10% DMSO show minimal stratification (Figure 2b) and also limited light-induced halide segregation (Figure S5, Supporting Information), whereas films prepared from DMF precursor solutions using 33% DMSO undergo halide segregation (Figure S7), similar to the results when using 20% DMSO.

*Halide Redistribution in Segregated Mixed-Halide Quasi-Two-Dimensional Perovskites*. When a segregated system is stored in the dark after being illuminated, the effects of halide demixing are typically reversed in 3D perovskites due to the entropy-driven restoration of the statistical composition as halide ions remix. Consequently, the PL blue-shifts to the original emission energy. For lower-dimensional systems, the structural nature of the perovskite was found to influence this behavior. For instance, in the case of a perovskite film prepared from DMF only (Figure 4b,c), the red-shifted emission reversed in the dark, and the PL peak approached the emission energy of the pristine film prior to illumination. The same behavior was observed in the 3D phase of a stratified quasi-2D perovskite (Figure 4d,e), where the PL peak blue-shifted from 1.63 to 1.78 eV over 14 h of storage in dark.

Remarkably, the peak assigned to the segregated *n* = 2 layered phases did not recover but rather remained at ∼2.18 eV throughout dark storage (Figure 4d,e). The same was the case for the segregated *n* = 1 phase, where the mildly red-shifted PL peak remained constant at the low emission energy. This observation indicates the absence of remixing of halides in *n* = 2 phases at room temperature, as observed for the *n* = 1 system. Perovskite films developed using other cosolvent compositions showed similar behavior (Figure S7) where the segregated 3D phase shows the typical signs of halide redistribution in the dark, whereas the *n* = 2 and *n* = 1 phases do not.

To induce a remixing of halide ions in lower-dimensional phases, the dark recovery of the PL of a halide-segregated film was tracked at elevated temperatures (Figure 5). Similar to the observations reported in Figure 4d, the peak corresponding to the 3D phase showed a blue-shift at room temperature, whereas the peaks for lower-dimensional phases did not. However, when maintained at 50 °C for 60 min, the PL peak related to a segregated *n* = 2 phase starts to decrease in intensity and thereafter, at a temperature of 70 °C, blue-shifts to develop the shoulder originally observed in pristine films. At higher temperatures, the PL signal is weaker and thus appears more noisy in the normalized spectra. Unnormalized data is reported in Figure S8. Interestingly, the PL peak corresponding to the *n* = 1 phase remains at a lower energy even at elevated temperatures, which indicates the absence of recovery.

**Figure 5:**
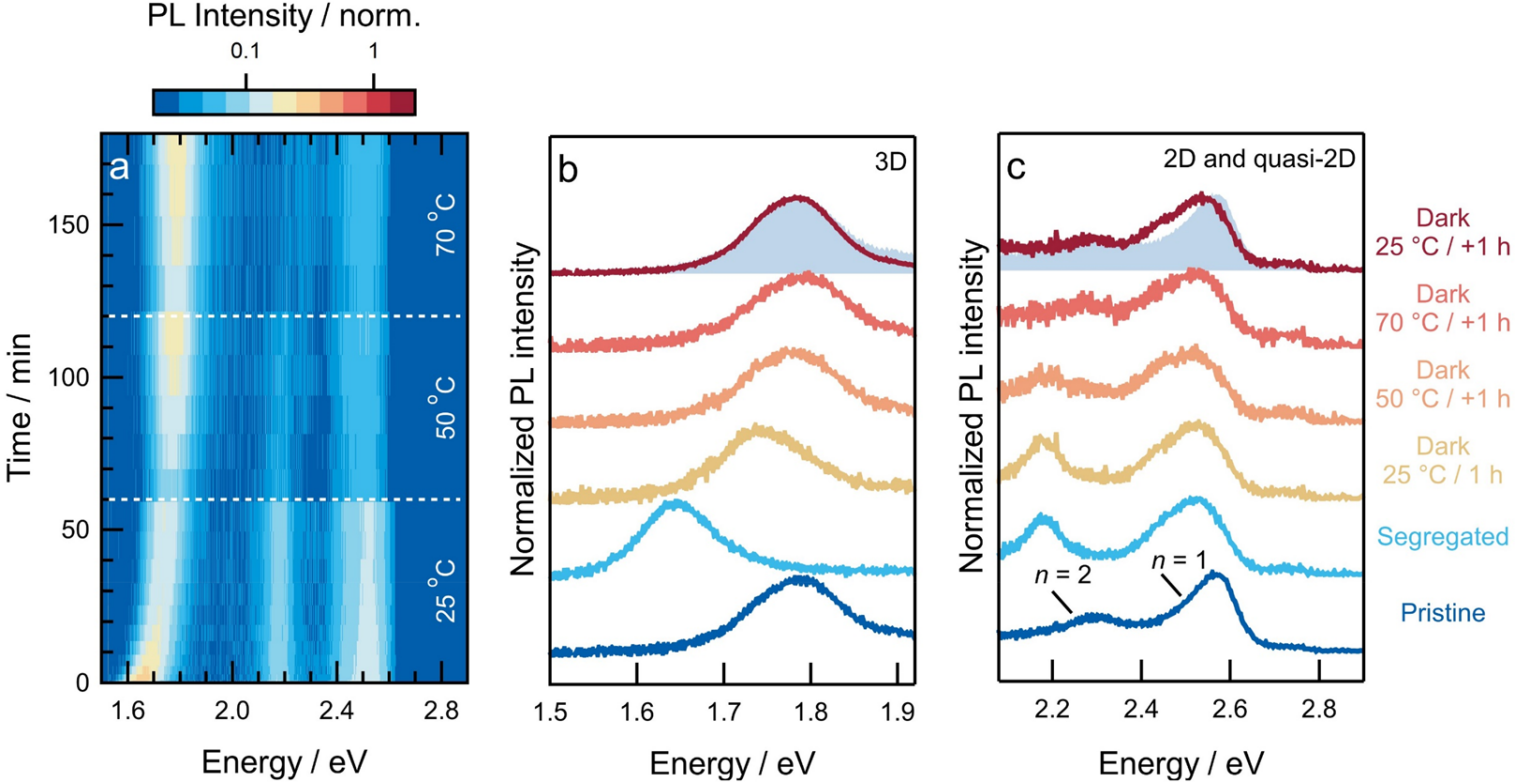
Dark recovery of halide segregation in lower-dimensional perovskites. (a) Normalized 2D plot of photoluminescence intensity versus photon energy recorded over time for a PEA_2_MA_3_Pb_4_(Br_0.33_I_0.67_)_13_ film during the dark recovery at different film temperatures after illumination for 2 h. (b,c) Photoluminescence spectra of 3D and 2D phases, normalized to the maximum peak intensity, at the start of the illumination (pristine), after 2 h of illumination (segregated), and after dark recovery at sequentially elevated temperatures (25, 50, and 70 °C) for 1 h each, followed by cooling to 25 °C for 1 h. The shaded areas in panels (b) and (c) correspond to the spectra of the pristine film and are shown to facilitate comparison with the spectra after recovery.

In conclusion, the dimensionality of perovskites in thin films plays an important role in their tendency to undergo light-induced halide segregation and, correspondingly, for halide ions to remix in the dark. Layered perovskites containing single-lead-halide octahedra sheets (*n* = 1) show limited signs of photoinduced halide segregation. The small red-shift observed in PL under illumination nevertheless shows that 2D systems are not completely immune to these processes. In a quasi-2D system with two conjoined octahedral sheets (*n* = 2), the experiments confirm the occurrence of halide segregation. However, the entropically driven redistribution of halides in the dark is still restricted and only occurs at elevated temperatures. Lastly, higher-*n* perovskites, for instance the quasi-3D perovskites prepared from a DMF-pure solution or the 3D phase when prepared from a DMF solution containing 20% DMSO, show the characteristic behavior of halide segregation under illumination and the subsequent recovery in the dark owing. However, a quasi-3D perovskite system still demonstrates higher photostability than a 3D perovskite formed in the stratified film.

Summarizing, mixed-halide PEA_2_MA_*n*–1_Pb_*n*_(Br_*x*_I_1–*x*_)_3*n*+1_ 2D perovskites demonstrate superior photostability when compared to 3D perovskites. This is, however, contingent on their structural properties and distribution of different layered phases in the film. Pure 2D layered systems containing a single sheet of lead halide octahedra (*n* = 1) are largely stable against photoinduced segregation of iodide and bromide ions. This is tentatively explained by the nonbinomial halide distribution revealed by ^207^Pb NMR, which increases the barrier to halide diffusion. Quasi-2D phases (*n* > 1), however, are more susceptible to light-induced halide demixing. When halide-segregated films are stored in dark, halide redistribution occurs in quasi-3D and 3D perovskites, signaled by a blue-shift in the PL spectrum. However, in layers containing fewer conjoined sheets (*n* = 2 or *n* = 1) halide redistribution is arrested because of higher ion-migration energy barriers, leading to a segregated phase that remains demixed over time.

## Supporting Information Available

Experimental Section, additional figures, and additional tables (PDF)
