# Supplementary material for: Light-Induced Halide Segregation in 2D and Quasi-2D Mixed-Halide Perovskites
Source: ACS Energy Lett. 2023 Mar 3;8(4):1662–70. doi: 10.1021/acsenergylett.3c00160 (PMC10111410; doi:10.1021/acsenergylett.3c00160)
Supplement: Supplementary file 1 — nz3c00160_si_001.pdf [file nz3c00160_si_001.pdf]

Supporting Information

Light-induced Halide Segregation in 2D and Quasi-2D Mixed-Halide Perovskites

*Kunal Datta,[1‡] Alessandro Caiazzo,[1‡] Michael A. Hope,[2] Junyu Li,[1] Aditya Mishra,[2] Manuel Cordova,[2] Zehua Chen,[3] Lyndon Emsley,[2] Martijn M. Wienk,[1] and René A. J. Janssen[1,4]**

[1] Molecular Materials and Nanosystems, Institute of Complex Molecular Systems, Eindhoven University of Technology, P.O. Box 513, 5600 MB, Eindhoven, The Netherlands

[2] Institut des Sciences et Ingénierie Chimiques, Ecole Polytechnique Fédérale de Lausanne, Lausanne CH – 1015, Switzerland

[3] Materials Simulation and Modelling and Center for Computational Energy Research, Department of Applied Physics, Eindhoven University of Technology, P.O. Box 513, 5600 MB, Eindhoven, The Netherlands

[4] Dutch Institute for Fundamental Energy Research, 5612 AJ Eindhoven, The Netherlands

[‡] These authors contributed equally.

1. Methods

Solution preparation and film fabrication

The precursor solutions were prepared by dissolving PEAI, MAI, PbI_2 in the ratio 2:$n-1$:n in DMF for n = 1 and in DMF/DMSO (0, 5%, 10%, 20%, or 33%) for $\langle n \rangle$ = 4, with overnight stirring at 60 °C. The concentration of the precursor solutions was 1 M in PbI_2. Glass substrates were cleaned by sonicating in acetone, scrubbing and sonicating in soap solution, rinsing in demineralized water, sonicating in 2-propanol, followed by 30 min UV-ozone treatment. 60 µL of the perovskite

precursor solution was spin coated at room-temperature at 5000 rpm for 45 s, followed by thermal annealing at 100 °C for 10 min.

Film characterization

UV-vis-NIR absorption spectra were recorded by using a PerkinElmer Lambda 1050 UV-vis-NIR spectrophotometer. Photoluminescence spectra were recorded by using an Edinburgh Instruments FLSP920 double-monochromator luminescence spectrophotometer. XRD patterns were recorded by using a Bruker 2D phaser (Cu Kα radiation, $\lambda = 1.5406$ Å): measurements were performed in the range 3-40° with a step size 0.02° and collection time of 1 s. 2D GIWAXS measurements were performed with a Ganesha 300XL+ system from JJ X-ray equipped with a Pilatus 300K detector (pixel side 172 μm × 172 μm). The X-ray source was a Genix 3D Microfocus sealed tube X-ray Cu-source with integrated monochromator. The wavelength used was 1.5406 Å. The detector moves in a vacuum chamber with sample-to-detector distance (SDD) varied between 0.115 m and 1.47 m depending on the configuration used, as calibrated using silver behenate (d_{001} = 58.380 Å). For GIWAXS, the SDD was 115.4 mm. The angle dependent 2D GIWAXS were conducted via controlling incident angle from 0.1-0.5° with a 0.1° interval.

For time- and illumination-dependent photoluminescence measurements, the samples were mounted into a custom-built sealed holder to maintain an inert atmosphere. To induce halide segregation, samples were illuminated with blue (Thorlabs M405L4, 405 nm), and where mentioned green (Thorlabs M530L3, 530 nm), light-emitting diodes at angles of about 45° to the surface normal. The green light was filtered by a 600 nm short-pass filter and the LEDs were driven by a Thorlabs DC4104 driver. During spectrum acquisition, the blue LED was used as the excitation source for photoluminescence measurements and the green LED was switched off. To track the photoluminescence behavior when the sample was stored in the dark, the blue LED was used intermittently (exposure time ~500 ms) as the excitation source and was otherwise switched

off. The photoluminescent light collected at 90° from the surface normal, filtered by a 645 nm long-pass filter, was focused onto an optical fiber connected to the spectrometer (Avantes Avaspec-2048x14) operated on a custom-built code in the LabVIEW environment. To measure photoluminescence behavior at elevated temperatures, the sample was loaded onto a heating stage (Linkam THMS 600) continuously flushed with nitrogen gas.

Solid-state NMR experiments were performed at room temperature on an 11.7 T Bruker Advance III spectrometer using 1.3 mm outer-diameter rotors. Isotropic ^{207}Pb spectra were obtained by summing the rows of sheared 2D PASS spectra, which separate the isotropic and anisotropic ^{207}Pb chemical shifts in a two-dimensional experiment. Five-pulse PASS spectra were acquired at 24 kHz MAS,[1] with a single rotor period for the PASS block, a radiofrequency amplitude of 250 kHz, eight increments in the indirect dimension, and a recycle delay of 0.1 s. Between 48,000 and 186,000 scans were acquired per increment, depending on the sample. Spectra were referenced to $Pb(NO_3)_2$ with an isotropic shift of −3490 ppm at room temperature. The chemical shielding calculations were performed on $[PbX_6]^{4-}$ octahedral clusters using the Amsterdam Density Functional (ADF) 2017 package, as previously reported for 3D perovskites.[2–4] The atomic positions were extracted for different local configurations from periodic DFT calculations, as reported in Ref.[17]. Relativistic effects were included through the zero-order regular approximation (ZORA) method at the spin–orbit level.[5–7] The BP86 functional with Grimme DFT-D3 dispersion correction and Becke and Johnson (BJ) damping was used along with ZORA/QZ4P all-electron basis sets.[8–11] Chemical shieldings were computed using the Gauge-including atomic orbital (GIAO) formalism.[12–16]

Additional Tables and Figures

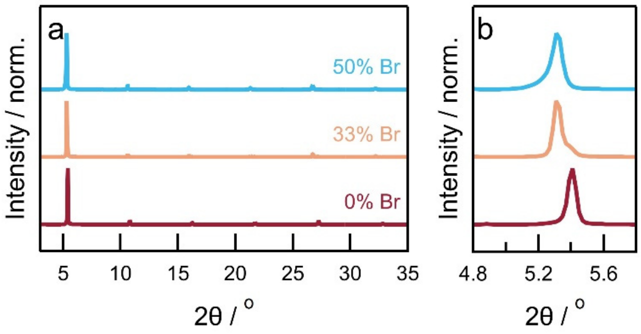

Figure S1. X-ray diffractograms of $PEA_2Pb(Br_xI_{1-x})_4$ for $x = 0.50$, 0.33, and 0.0.

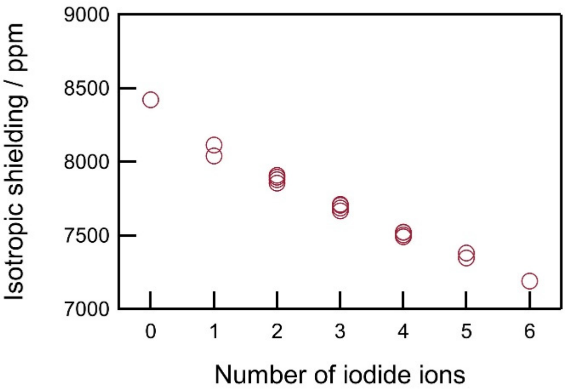

Figure S2. Calculated isotropic shieldings for different $[PbI_aBr_{6-a}]^{4-}$ configurations as function of the number of iodine atoms, a.

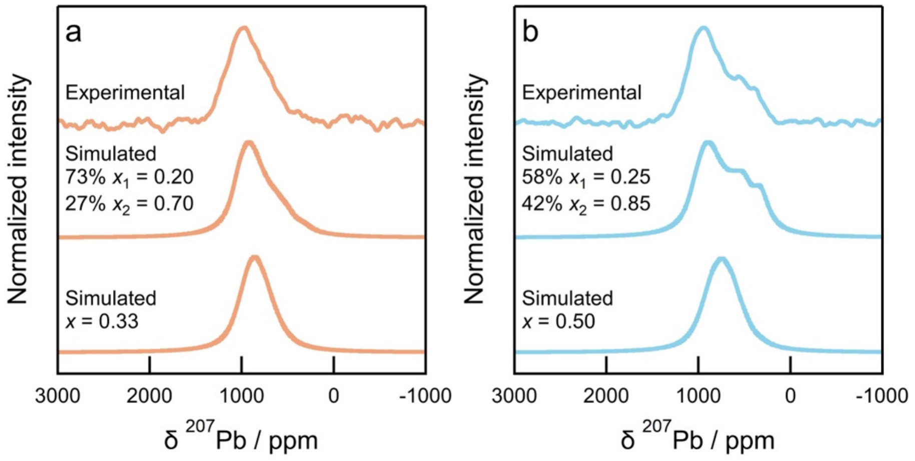

Figure S3. Experimental and simulated isotropic ^{207}Pb spectra of $PEA_2Pb(Br_xI_{1-x})_4$ for (a) $x = 0.33$ and (b) $x = 0.50$. The simulated spectra are obtained by summation of gaussians centered at the calculated isotropic shifts for each configuration (see Table S1), each with a full-width-at-half-maximum of 220 ppm taken from the experimental spectrum of PEA_2PbI_4. In the bottom spectra, the weights of each configuration are given by a single binomial distribution of halides within the nominal composition of the sample (x). The middle spectra represent a model where the sample is assumed to be comprised of two sets of $Pb(Br_xI_{1-x})_6$ octahedra with different average compositions (x_1, x_2) in different proportions, that averaged together give the nominal sample composition (for (a), $x = 73\% \times 0.20 + 27\% \times 0.70 = 0.33$; for (b), $x = 58\% \times 0.25 + 42\% \times 0.85 = 0.5$). The weights of each configuration are calculated from the binomial distribution for the two sets then combined via the relative proportions. The weights of each configuration are calculated from the binomial distribution for the two regions then combined via the relative proportions. The experimental spectra (top) are as in Figure 1b. Although this simple model does not exactly match experiment, which could also result from limitations in the chemical shift calculations, it nevertheless supports the hypothesis of halide clustering. Importantly, the simulated spectra with a random halide distribution are not consistent with the experimental results.

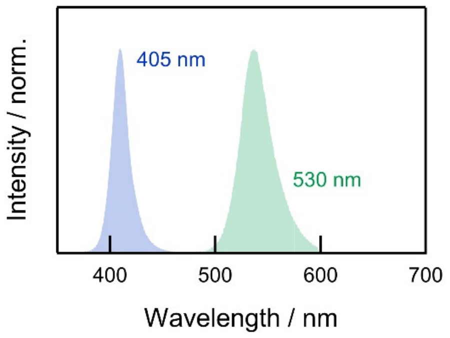

Figure S4. Emission spectra of blue (405 nm) and green (530 nm) LED sources.

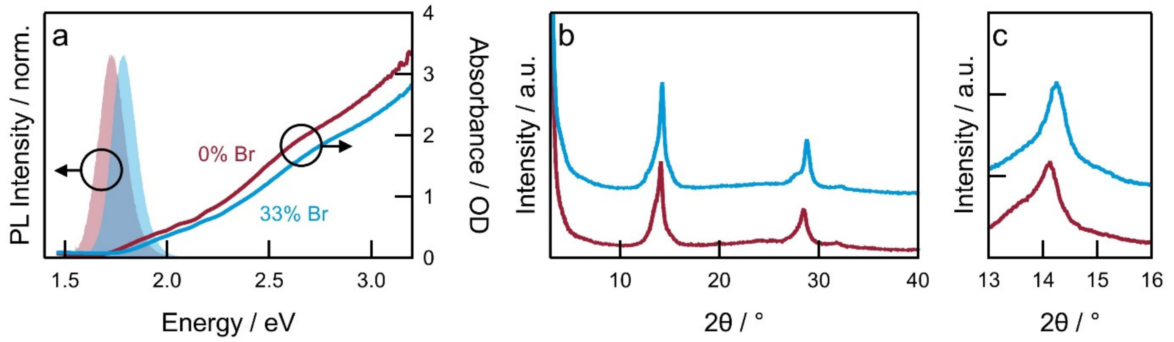

Figure S5. a UV-vis-NIR absorption and photoluminescence spectra of $PEA_2MA_3Pb_4(Br_xI_{1-x})_{13}$ perovskite thin films with x = 0 and x = 0.33 prepared from a pure DMF precursor solution. **b-c** Corresponding X-ray diffractograms.

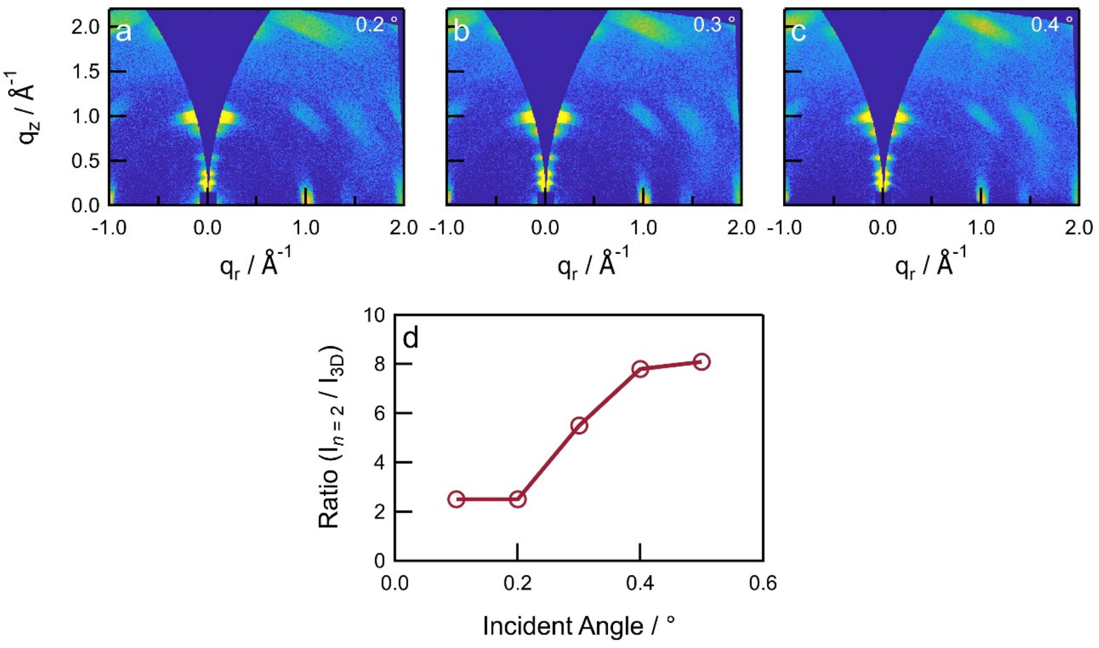

Figure S6. a-c AR-GIWAXS patterns for $PEA_2MA_3Pb_4(I_{0.67}Br_{0.33})_{13}$ (cast from DMF with 20% DMSO) measured at incident angles of (a) 0.2°, (b) 0.3°, and (c) 0.4°. **d** Ratio of peak intensity ($I_{n=2}/I_{3D}$) as a function of incident angle.

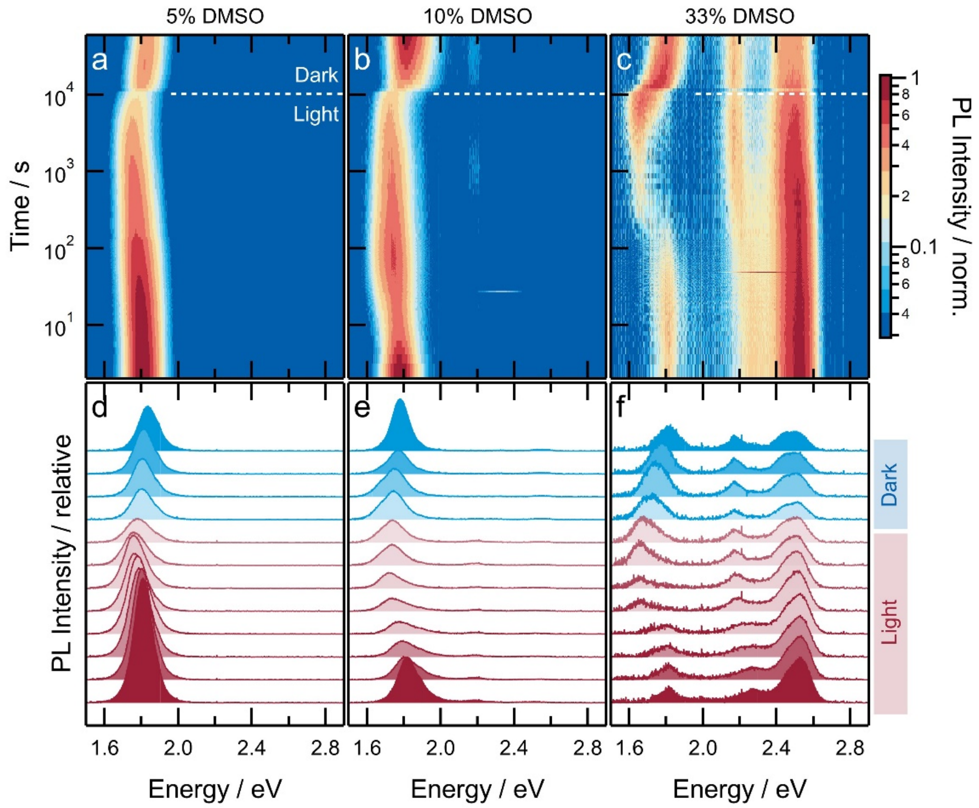

Figure S7. a-c Normalized 2D plots of the photoluminescence intensity versus photon energy of $PEA_2MA_3Pb_4(Br_{0.33}I_{0.67})_{13}$ films recorded over time under continuous excitation (~3 h) followed by storage in the dark (~14 h). **d-f** Corresponding photoluminescence spectra. Films were prepared from DMF with 5% DMSO (a,d), 10% DMSO (b,e), and 33% DMSO (c,f) in the precursor solution.

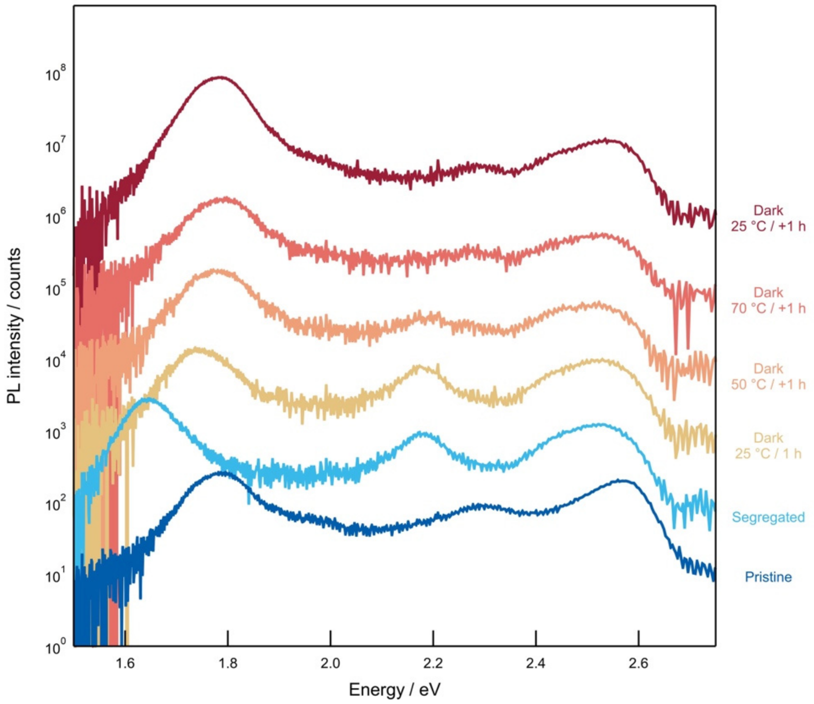

Figure S8. Unnormalized PL spectra for the data displayed in Figure 5. The data is stacked and plotted in log scale for clarity.

Table S1. The 18 possible configurations of $[PbI_aBr_{6-a}]$ octahedra for $n = 1$ layered mixed-halide $PEA_2Pb(Br_xI_{1-x})_3$ perovskites, assuming the two axial sites are equivalent, the four equatorial sites are equivalent, but the axial and equatorial sites are inequivalent. The probability of each configuration for an $x = 0.5$ sample with a random binomial distribution is shown. The calculated isotropic shieldings are converted to isotropic chemical shifts by interpolating between the experimental shifts of the pure bromide and pure iodide samples ($\delta = 5147.4 - 0.57266\sigma$).

**Iodines**	**Axial Iodines**	**Equatorial Iodines**		**Degeneracy**	**Probability (x = 0.5)**	**Shielding σ /ppm**	**Shift δ /ppm**
0	0	0		1	1.6%	8421	325
1	1	0		2	3.1%	8041	543
1	0	1		4	6.3%	8115	500
2	2	0		1	1.6%	7855	649
2	1	1		8	12.5%	7879	636
2	0	2	cis	4	6.3%	7909	618
2	0	2	trans	2	3.1%	7897	625
3	2	1		4	6.3%	7667	757
3	0	3		4	6.3%	7713	731
3	1	2	cis	8	12.5%	7705	735
3	1	2	trans	4	6.3%	7685	746
4	0	4		1	1.6%	7522	840
4	1	3		8	12.5%	7522	840
4	2	2	cis	4	6.3%	7505	850
4	2	2	trans	2	3.1%	7493	857
5	1	4		2	3.1%	7380	921
5	2	3		4	6.3%	7347	940
6	2	4		1	1.6%	7190	1030

Supplementary References

(1) Antzutkin, O. N.; Shekar, S. C.; Levitt, M. H. Two-Dimensional Sideband Separation in Magic-Angle-Spinning NMR. *J. Magn. Reson. A* **1995**, *115* (1), 7–19.

(2) te Velde, G.; Bickelhaupt, F. M.; Baerends, E. J.; Fonseca Guerra, C.; van Gisbergen, S. J. A.; Snijders, J. G.; Ziegler, T. Chemistry with ADF. *J. Comput. Chem.* **2001**, *22* (9), 931–967.

(3) Askar, A. M.; Karmakar, A.; Bernard, G. M.; Ha, M.; Terskikh, V. v.; Wiltshire, B. D.; Patel, S.; Fleet, J.; Shankar, K.; Michaelis, V. K. Composition-Tunable Formamidinium Lead Mixed Halide Perovskites via Solvent-Free Mechanochemical Synthesis: Decoding the Pb Environments Using Solid-State NMR Spectroscopy. *J. Phys. Chem. Lett.* **2018**, *9* (10), 2671–2677.

(4) Karmakar, A.; Askar, A. M.; Bernard, G. M.; Terskikh, V. v.; Ha, M.; Patel, S.; Shankar, K.; Michaelis, V. K. Mechanochemical Synthesis of Methylammonium Lead Mixed-Halide Perovskites:

Unraveling the Solid-Solution Behavior Using Solid-State NMR. *Chem. Mater.* **2018**, *30* (7), 2309–2321.

(5) van Lenthe, E.; Baerends, E. J.; Snijders, J. G. Relativistic Total Energy Using Regular Approximations. *J. Chem. Phys*. **1994**, *101* (11), 9783–9792.

(6) van Lenthe, E. Geometry Optimizations in the Zero Order Regular Approximation for Relativistic Effects. *J. Chem. Phys.*. **1999**, *110* (18), 8943–8953.

(7) van Lenthe, E.; Baerends, E. J.; Snijders, J. G. Relativistic Regular Two-Component Hamiltonians. *J. Chem. Phys*. **1993**, *99* (6), 4597–4610.

(8) Perdew, J. P. Density-Functional Approximation for the Correlation Energy of the Inhomogeneous Electron Gas. *Phys. Rev. B*. **1986**, *33* (12), 8822–8824.

(9) Becke, A. D. Density-Functional Exchange-Energy Approximation with Correct Asymptotic Behavior. *Phys. Rev. A* **1988**, *38* (6), 3098–3100.

(10) Grimme, S.; Ehrlich, S.; Goerigk, L. Effect of the Damping Function in Dispersion Corrected Density Functional Theory. *J. Comput. Chem*. **2011**, *32* (7), 1456–1465.

(11) van Lenthe, E.; Baerends, E. J. Optimized Slater-Type Basis Sets for the Elements 1-118. *J. Comput. Chem*. **2003**, *24* (9), 1142–1156.

(12) Schreckenbach, G.; Ziegler, T. The Calculation of NMR Shielding Tensors Based on Density Functional Theory and the Frozen-Core Approximation. *Int. J. Quantum Chem*. **1996**, *60* (3), 753–766.

(13) Wolff, S. K.; Ziegler, T. Calculation of DFT-GIAO NMR Shifts with the Inclusion of Spin-Orbit Coupling. *J. Chem. Phys.* **1998**, *109* (3), 895–905.

(14) Wolff, S. K.; Ziegler, T.; van Lenthe, E.; Baerends, E. J. Density Functional Calculations of Nuclear Magnetic Shieldings Using the Zeroth-Order Regular Approximation (ZORA) for Relativistic Effects: ZORA Nuclear Magnetic Resonance. *J. Chem. Phys.* **1999**, *110* (16), 7689–7698.

(15) Schreckenbach, G.; Ziegler, T. The Calculation of NMR Shielding Tensors Based on Density Functional Theory and the Frozen-Core Approximation. *Int. J. Quantum Chem*. **1996**, *60* (3), 753–766.

(16) Schreckenbach, G.; Ziegler, T. Calculation of NMR Shielding Tensors Based on Density Functional Theory and a Scalar Relativistic Pauli-Type Hamiltonian. The Application to Transition Metal Complexes. *Int. J. Quantum Chem*. **1997**, *61* (6), 899–918.

(17) Chen, Z.; Haibo, X.; Geert B.; Bobbert P. A.; Tao S. Thermodynamic Origin of the Photostability of the Two-Dimensional Perovskite $PEA_2Pb(I_{1\text{-}x}Br_x)_4$. *ACS Energy Lett.* **2023**, *8* (2), 943–949.
